# A Novel Atypical PKC-Iota Inhibitor, Echinochrome A, Enhances Cardiomyocyte Differentiation from Mouse Embryonic Stem Cells

**DOI:** 10.3390/md16060192

**Published:** 2018-06-02

**Authors:** Hyoung Kyu Kim, Sung Woo Cho, Hye Jin Heo, Seung Hun Jeong, Min Kim, Kyung Soo Ko, Byoung Doo Rhee, Natalia P. Mishchenko, Elena A. Vasileva, Sergey A. Fedoreyev, Valentin A. Stonik, Jin Han

**Affiliations:** 1 National Research Laboratory for Mitochondrial Signaling, Department of Physiology, Department of Health Sciences and Technology, BK21 Plus Project Team, Cardiovascular and Metabolic Disease Center, Inje University College of Medicine, Busan 47392, Korea; estrus74@gmail.com (H.K.K.); drswcho@hanmail.net (S.W.C.); hjheo0303@gmail.com (H.J.H.); shjeong96@gmail.com (S.H.J.); glucose1193@gmail.com (M.K.); kskomd@paik.ac.kr (K.S.K.); bdrhee@hanmail.net (B.D.R.); 2 Department of Integrated Biomedical Science, Inje University College of Medicine, Busan 47392, Korea; 3 Division of Cardiology, Department of Internal Medicine, Inje University College of Medicine, Seoul Paik Hospital, Seoul 04551, Korea; 4 G.B. Elyakov Pacific Institute of Bioorganic Chemistry, Far-Eastern Branch of the Russian Academy of Science, Vladivostok 690022, Russia; mischenkonp@mail.ru (N.P.M.); vasilieva_el_an@mail.ru (E.A.V.); fedoreev@piboc.dvo.ru (S.A.F.); stonik@piboc.dvo.ru (V.A.S.)

**Keywords:** echinochrome A, embryonic stem cell, cardiomyocyte differentiation, PKC-iota

## Abstract

Echinochrome A (EchA) is a marine bioproduct extracted from sea urchins having antioxidant, antimicrobial, anti-inflammatory, and chelating effects, and is the active component of the clinical drug histochrome. We investigated the potential use of Ech A for inducing cardiomyocyte differentiation from mouse embryonic stem cells (mESCs). We also assessed the effects of Ech A on mitochondrial mass, inner membrane potential (Δψm), reactive oxygen species generation, and levels of Ca^2+^. To identify the direct target of Ech A, we performed in vitro kinase activity and surface plasmon resonance binding assays. Ech A dose-dependently enhanced cardiomyocyte differentiation with higher beating rates. Ech A (50 μM) increased the mitochondrial mass and membrane potential but did not alter the mitochondrial superoxide and Ca^2+^ levels. The in vitro kinase activity of the atypical protein kinase C-iota (PKCι) was significantly decreased by 50 μM of Ech A with an IC_50_ for PKCι activity of 107 μM. Computational protein-ligand docking simulation results suggested the direct binding of Ech A to PKCι, and surface plasmon resonance confirmed the direct binding with a low K_D_ of 6.3 nM. Therefore, Ech A is a potential drug for enhancing cardiomyocyte differentiation from mESCs through direct binding to PKCι and inhibition of its activity.

## 1. Introduction

Cardiovascular diseases are the leading cause of death worldwide. Significant myocardial damage can manifest as structural changes and decreased functional capacity of the heart, ultimately resulting in heart failure [1]. Unlike other human cell types, adult cardiomyocytes rarely proliferate or regenerate [2]. Therefore, many researchers have sought to develop efficient methods for cardiac regeneration after myocardial injury using cardiac stem cells [1,3,4]. However, many challenges still remain before cardiac stem cell therapy becomes a clinically applicable strategy for cardiac regeneration [4]. Among these challenges, obtaining a large number of purified cardiomyocytes from stem cells is the most important one [2].

Pluripotent stem cells (PSCs), including embryonic stem cells and induced pluripotent stem cells, are attractive cellular sources for cardiac stem cell therapy [5]. Various chemical compounds have been studied as potential regulators of the differentiation of PSCs into cardiac lineages, and most of these chemical compounds have been related to diverse signaling pathways involving the bone morphogenetic protein, transforming growth factor, activin, nodal, Wnt, rho-associated coiled-coil kinase, and fibroblast growth factor [5,6]. However, cardiogenic molecules extracted from natural sources remain to be investigated.

Echinochrome A (Ech A) is a bioactive red pigment isolated from sea urchins [7,8]. Histochrome, a drug form of Ech A, has shown cardioprotective effects against ischemia/reperfusion injury through its antioxidant and anti-inflammatory activities [9,10]. Our recent reports described various novel biological roles of Ech A, including enhancing mitochondria biogenesis via activation of the PGC1-α pathway [11], protecting mitochondrial function against cardiotoxic drugs [12], inhibiting acetylcholinesterase activity, and modulating the activity of cardiac sarcoplasmic reticulum Ca^2+^-ATPase [13]. Together, these previous studies suggest that Ech A is a candidate molecule to promote cardiac regeneration. In the present study, we showed the promotion of cardiomyocyte differentiation from mouse embryonic stem cells (mESCs) induced by Ech A, and the potential mechanism of Ech A-induced cardiac differentiation.

## 2. Results

### 2.1. Ech A Enhances Cardiomyocyte Differentiation from mESCs

**Figure 1:**
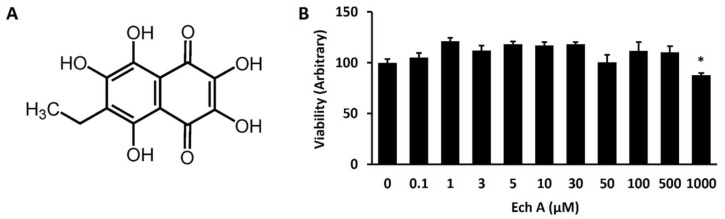
The chemical structure and cell viability effect of echinochrome A (Ech A). (**A**) The chemical structure of Ech A (6-ethyl-2,3,5,7,8-pentahydroxy-1,4-naphthoquinone; FW = 266.2). (**B**) The cell viability according to the dose of Ech A. Each group, *n* = 3–5.

**Figure 2:**
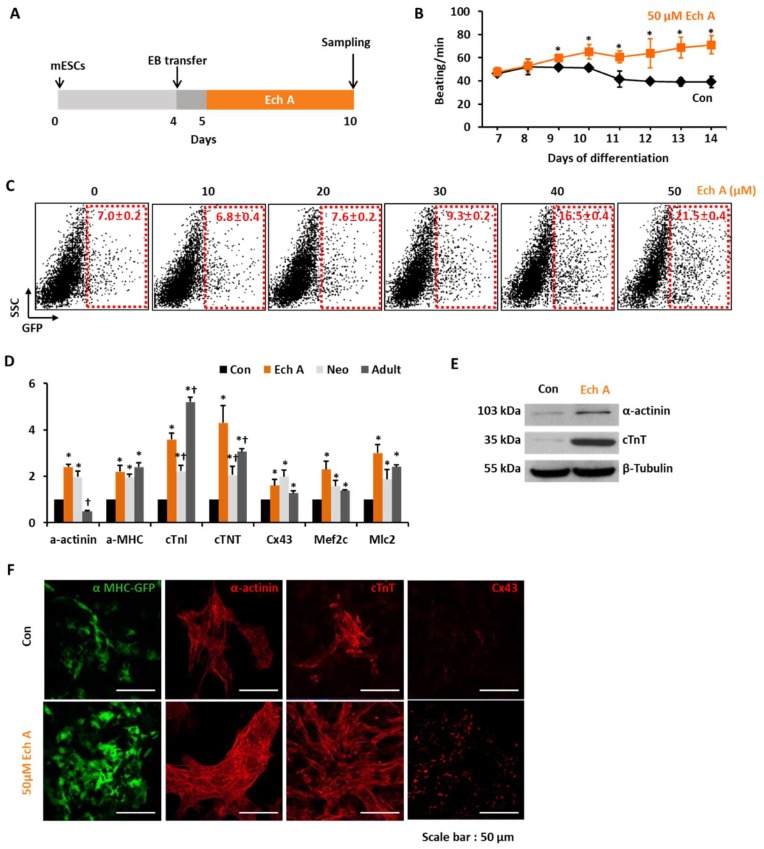
Ech A enhances cardiomyocyte differentiation from mouse embryonic stem cells (mESCs). (**A**) The protocol for the differentiation of cardiomyocytes from the mESCs using the hanging drop method to produce differentiated embryoid bodies (EBs). (**B**) The beating number per minute (BPM) of EBs during differentiation. Each group, *n* = 3. * *p* < 0.05 (**C**) Representative FACS analyses and the percentage of mESC-derived αMHC^+^ cardiomyocyte-like cells according to the dose of Ech A. Each group, n = 3. (**D**) Relative mRNA expression levels of cardiomyocyte specific genes in nontreated control (Con), Ech A treatment (Ech), heart tissue from neonatal (7 day, Neo) or adult mice (10 weeks, Adult). Each group, *n* = 3. * *p* < 0.05 vs. Con, **^†^***p* < 0.05 vs. Ech A. The mRNA level of glyceraldehyde-3-phosphate dehydrogenase (GAPDH) was used as an internal control. (**E**) Western blot analyses for the expression of cardiomyocyte specific proteins incubated with the control vehicle (Con) or 50 µM of Ech A. (**F**) Images displaying αMHC-GFP^+^, α-actinin^+^, cTnT^+^, and Cx43^+^ cells incubated with Con or 50 µM of Ech A (scale bars, 50 μm). SCC: side scatter cell, cTnI: cardiac troponin I, cTNT: cardiac troponin T, Cx43: connexin 43, Mef2c: Myocyte Enhancer Factor 2C, Mlc2: Myosin light chain 2.

Ech A is a water-insoluble compound extracted from the sea urchin *Scaphechinus mirabilis* and the active substance (P N002362/01) in the drug histochrome [12]. The chemical structure of Ech A is shown in Figure 1A. Ech A treatment up to 500 µM was not significantly toxic to EMG7 mESCs, however, Ech A reduced cell viability up to 87% of the control at a dose of 1mM (Figure 1B). For monitoring cardiomyocyte differentiation, we used EMG7 mESCs, which have a transgene consisting of cardiac-specific α-myosin heavy chain (αMHC) promoter-driven enhanced green fluorescent protein (GFP) [14]. Five days after the start of differentiation, 50 µM Ech A was treated to differentiating EBs (Figure 2A). Notably, 50 µM Ech A significantly increased the number of beating cardiomyocyte colonies in differentiating EBs, with a higher beating rate compared with the control vehicle-treated EBs (Movie 1 and Figure 2B). Ech A enhanced αMHC-GFP^+^ cardiomyocyte differentiation in a dose dependent manner, showing maximum efficacy at 50 µM (Figure 2C). Next, we compared the mRNA expression levels of genes related to cardiomyocyte differentiation (α-actinin, αMHC, cTnI, cTnT, connexin 43, and MLC2) between a non-treated control, and the Ech A treated group, 7 days neonatal mice hearts and 10 weeks adult mice hearts. The mRNA level of glyceraldehyde-3-phosphate dehydrogenase (GAPDH) was used as an internal control. The mRNA expression level of marker genes in the Ech A group was similar with the neonatal and adult mice ventricle than that of the control group (Figure 2D). Interestingly, only two genes, α-actinin and cTnI, showed significant different expression levels between the neonatal and adult heart. The expression of two genes in the Ech A group were more close to the neonatal heart. These results suggested that the Ech A treated differentiated cardiomyocyte-like cell is immature or neonatal, like cardiomyocyte.

By western blotting and immunocytochemistry, we found that 50 µM Ech A also significantly increased α-actinin, cTnT, and connexin 43 protein levels compared with the control (Figure 2E,F).

### 2.2. Ech A Enhances Cardiomyocyte Differentiation Through Direct Binding to Atypical PKCι and Inhibition of Its Activity

**Figure 3:**
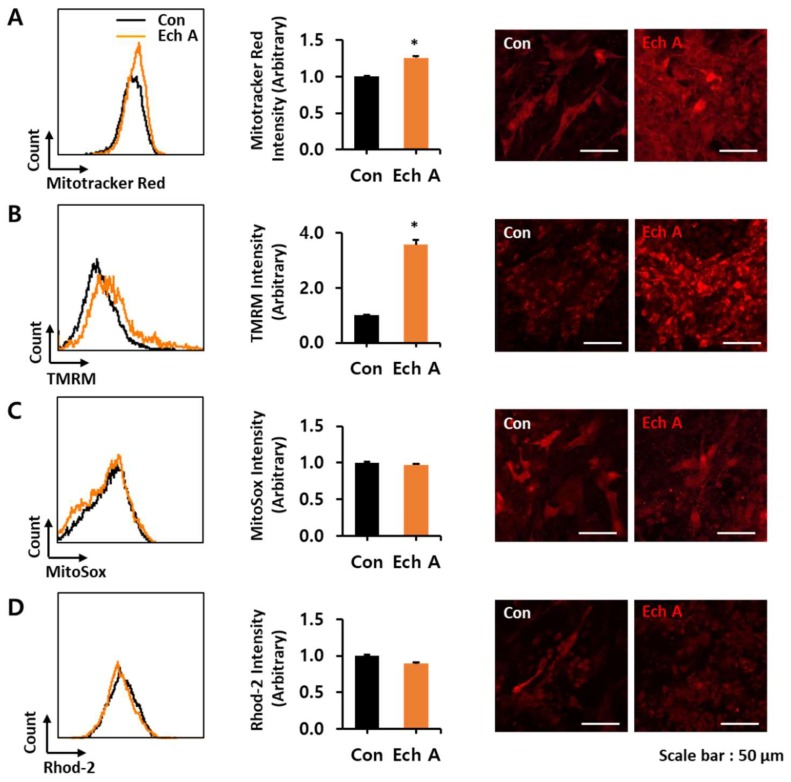
Measurement of mitochondrial functions in differentiating embryoid bodies (EBs) after Ech A treatment. Representative FACS analyses of fluorescence intensities (**left**), the percentage of relative mean fluorescence intensities (**middle**), and matched confocal microscope live cell images (**right**, scale bars, 50 μm) of (**A**) Mitotracker™, (**B**) mitochondria membrane potential fluorescence probe, TMRM, (**C**) Mitosox™, and (**D**) Rhod-2, which were treated with the control vehicle (Con) or 50 µM of Ech A. Each group, *n* = 3. * *p* < 0.05 vs non-treated control vehicle.

To confirm the effect of Ech A on mitochondria during cardiomyocyte differentiation, we measured the fluorescence intensity and membrane potential (Δψm) of the mitochondria using the Mitotracker™ Red and tetramethylrhodamine methyl ester (TMRM) fluorescent dye, respectively. Mitochondrial superoxide was measured using the MitoSox™ fluorescent dye and mitochondrial Ca^2+^ measurements used the Rhod-2AM fluorescent dye. Ech A significantly increased the mitochondrial mass and Δψm compared with the control, but did not affect the mitochondrial superoxide and Ca^2+^ levels during cardiomyocyte-like cell differentiation (Figure 3A–D). Taken together, these results showed that Ech A did not alter the mitochondrial function during cardiomyocyte-like cell differentiation.

**Figure 4:**
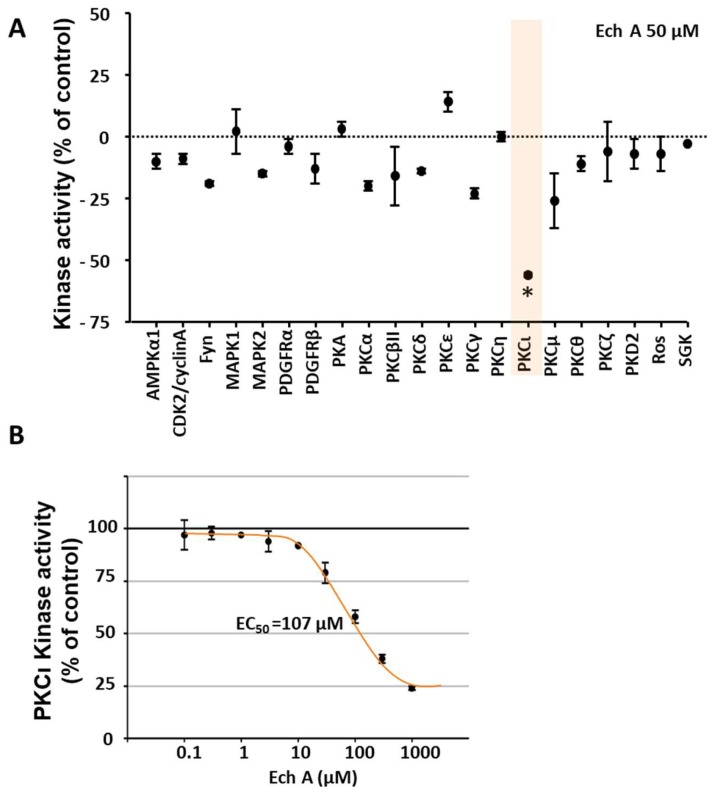
The in vitro kinase activity assay identified PKCι as a direct target kinase of Ech A. (**A**) The in vitro activity assay of selected kinases in the presence of 50 µM of Ech A. (**B**) The dose-dependent inhibition of PKCι kinase activity using 0–1000 μM of Ech A. * *p* < 0.05 vs vehicle control (Student’s *t*-test) (*n* = 3).

We then performed an in vitro kinase activity assay in differentiating EBs to identify the target of Ech A. We first performed kinase activity screening with a fixed concentration of 50 μM to find the target kinase of Ech A (Figure 4A). The result showed the significant inhibitory effect of Ech A to atypical PKC-iota activity (PKCι) among the tested kinases. Since the primary screening study used a single dose treatment, the relative inhibitory value could be inaccurate. To confirm the screening result, we further performed a multi-dose half-maximal inhibitory (IC_50_) study of Ech A on PKCι, which provided clear dose-dependent inhibitory effect of Ech A on PKCι. The IC_50_ of Ech A on PKCι activity was 107 μM, which was higher than primary screening result (Figure 4B).

**Figure 5:**
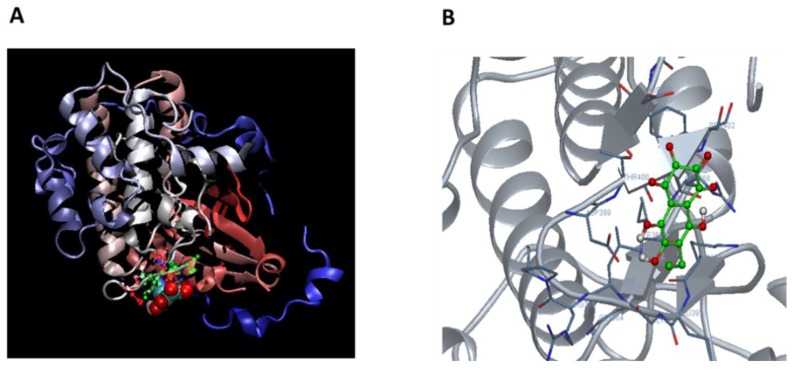
Computer simulated Ech A-PKCι docking model. (**A**) A three-dimensional (3D) model of Ech A-PKCι (PDB:3A8W) docking simulation was generated by using the online DockingServer site (https://www.dockingserver.com). (**B**) Magnified 3D structure of the Ech A-PKCι binding site.

**Figure 6:**
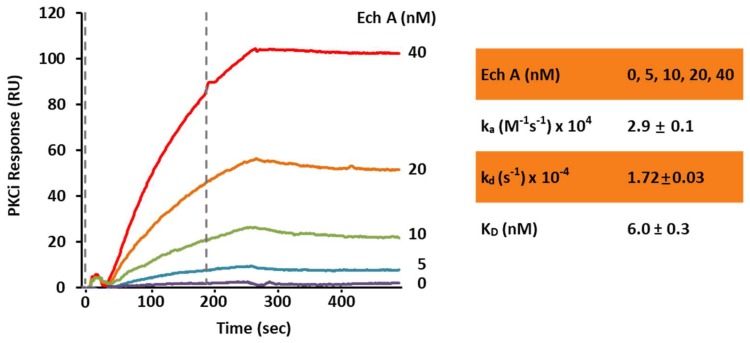
In vitro surface plasmon resonance binding assay for PKCι and Ech A. The PKCι binding response to Ech A was measured in the range of 0–40 nM of Ech A. The flow rate was 30 μL/minute. The association and dissociation times were 3 min each. The calculated binding constants are shown at the bottom. k_a_: Association rate, k_d_: Dissociation rate, K_D_ (Affinity) = k_d_/k_a_ (*n* = 3).

To confirm the direct binding of Ech A and PKCι, we performed a computational protein-ligand docking simulation and surface plasmon resonance binding assay. Computational protein-ligand docking simulation results suggested a probable binding site of Ech A to PKCι kinase domain residues 392–402 (KEGLRPGDTTS) by polar, hydrophobic and hydrogen bond interactions (Figure 5A,B; Table 1) and the surface plasmon resonance assay confirmed the direct binding of Ech A to PKCι with a low K_D_ value of 6.3 nM (Figure 6).

## 3. Discussion

Various cardiogenic compounds that modulate diverse signaling pathways have been developed to induce the differentiation of cardiomyocytes from PSCs [5]; however, natural cardiogenic resources with similar action have rarely been reported. In the present study, we demonstrated the cardiogenic potential of Ech A, a natural pigment isolated from sea urchins, and elucidated its action mechanism using EBs as a model system. Previous studies reported that Histochrome, a drug form of Ech A, had a cardioprotective effect involving antioxidant and anti-inflammatory mechanisms [10]. In addition, our group previously reported that Ech A had diverse biological effects involving the enhancement of mitochondrial functions [11,12]. In the present study, we firstly discovered the cardiomyogenic effect of Histochrome and its novel molecular target—the PKC-iota—which may extend the application of Histochrome not just as a cardioprotective drug, but for the cardiomyogenic drugs combined with stem cell therapy as well.

Ech A has no significant cytotoxicity at doses up to 500 μM. The 50 μM of Ech A treatment significantly enhanced the beating rate of the differentiated colony (71 ± 4.5/min on day 14) than the untreated control (52 ± 1.0/min on day 10). The tendency of increasing BPM is kept until 14 days in the Ech A group (Figure 2B). The heart rate of the mouse varied from 500/min to 700/min depending on the time of the day, environmental factors, and mice spices. However, because of the environmental differences between the in vitro cell line and the in vivo heart condition, the heart rates of the control and Ech A treated groups were significantly different from those of the actual in vivo heart.

Mitochondrial function and signaling pathways have been investigated as a key regulator of cardiac differentiation and regeneration [15,16,17]. During differentiation from ESCs to cardiomyocytes, mitochondrial morphology changes dramatically, and mitochondrial biogenesis and oxidative metabolism increase significantly [16,18]. These changes could be considered a consequence, rather than a cause, of cardiomyocyte-like cell differentiation, which simply reflect the increasing contraction and metabolic demands of cardiomyogenesis [14]. However, several studies reported that mitochondrial function and maturation does in fact play a role in controlling cardiomyocyte differentiation from PSCs [15,16]. Our group previously reported that mitochondrial pyruvate dehydrogenase phosphatase 1 regulates the early differentiation of cardiomyocytes from mESCs [19], and that a combination of cyclosporin A with antioxidants synergistically promotes cardiomyocyte differentiation from PSCs by modulating the mitochondrial permeability transition pore and redox signaling [14]. In the present study, we also investigated the effects of Ech A on mitochondria in differentiating EBs. Ech A significantly increased the mitochondrial mass and Δψm compared to control, but did not affect mitochondrial superoxide and Ca^2+^ levels. These findings suggested that Ech A did not affect mitochondrial function in differentiating EBs, and that the increase in mitochondrial mass and Δψm was a consequence of cardiomyocyte differentiation from EBs.

In the present study, we also showed that the cardiogenic potential of Ech A is realized via direct binding and inhibition of PKCι activity. PKCs are serine-threonine kinases that regulate various cellular processes. These kinases are classified into the three subtypes of conventional, novel, and atypical PKCs. Several previous studies reported that some PKC subtypes regulate embryonic development and PSC self-renewal and differentiation [20,21,22]. Among the PKC subtypes, a recent study reported that atypical PKCι controls stem/progenitor cell expansion via regulation of the Notch pathway [21]. Notably, knockdown of PKCι in EBs resulted in increased expression of the cardiac-specific markers Isl1, Nkx2.5, and α-actinin when compared to control EBs [21]. Similarly, our data also showed that the kinase activity of PKCι significantly decreased in a dose-dependent manner by in vitro Ech A treatment. These results suggested that the inhibition of atypical PKCι might be an important driver for cardiac differentiation of PSCs. In addition to an in vitro cell-free kinase activity assay, we further tested the direct binding of Ech A and PKCι using computational modeling and a surface plasmon resonance binding assay. The docking simulations suggested direct binding of Ech A to the PKCι kinase domain at amino acids 390–420 (Figure 5, Table 1). In the study of Pillai et al. [23], the investigators developed a novel PKCι inhibitor ICA-1 and performed the docking site simulation between ICA-1 and PKCι kinase domain by using PDB code 1ZRZ, similar with our present study. The ICA-1 was proposed to bind at the 469–475 amino acid residue of the PKCι kinase domain, while Ech A is predicted to bind at the 390–420 amino acid residue of it. Both predicted sites are not located at the conserved ATP binding pocket of the PKC isoforms, suggesting the PKCι isoform specific inhibition potential of both drugs [23]. Because PKCι and PKC-zeta (PKCζ) share 84% homology in amino acid sequences in their kinase domain [23], the PKCι specific inhibitory effect of Ech A could be supported by our kinase assay results in Figure 4A, which showed a significant difference between PKCι and other isoforms, especially PKCζ. However, the molecular biology based confirmation of the binding between Ech A and PKCι kinase domain is necessary to develop the Ech A as a PKCι inhibiting drug.

To solidify the evidence of the ligand-protein physical interaction, surface plasmon resonance showed the dose-dependent increase of the binding response signal between PKCι and Ech A (Figure 6). The low K_D_ of 6.03 nM suggested a high affinity between these two components. Moreover, the Ech A-PKCι binding did not dissociate during a 3-min time period. Interestingly, although the affinity of Ech A to PKCι is very high, the sufficient dose for the inhibition is relatively high as IC_50_ = 107 μM in the cell free in vitro system. These affinity and efficiency should be tested more detailed in the in vivo system in order to develop Ech A as a PKCι targeting drug.

Our results collectively demonstrated the effect of Ech A on cardiomyogenesis, and identified an atypical PKCι as a direct binding target. Although the differentiated EBs and cells in the present study showed cardiomyocytes-like characteristics in the beating function and the expression of various cardiac specific markers, the in vitro differentiated “cardiomyocyte-like cells” still differed from real cardiomyocytes in the integrity of various ion channels, intracellular Ca^2+^ modulations, cell to cell connectivity, and so on. Therefore, further studies must elucidate (i) the regulatory mechanism of Ech A during the enhancement of cardiomyogenesis, and (ii) the effect of Ech A on the maturation or optimization of “cardiomyocyte-like cells” to clinically applicable cardiomyocytes.

In addition to the effects on cardiomyogenesis, PKCι inhibition has been proposed as a novel therapeutic target for acute pain syndrome [24], inflammatory disorders [25], and various cancers including prostate cancer [26], neuroblastoma [23], glioblastoma [27,28], pancreatic cancer [29], and melanoma [30]. The novel PKCι inhibitor, Ech A, therefore has great potential for a wide range of clinical applications.

As a limitation of our study, the efficacy of Ech A is relatively lower (21% of total cell population) than established combined methods, which had improved the efficiency of stem cell differentiation to cardiomyocyte around 70–95% of the total population. According to these protocols, cardiac specification and differentiation processes are finely regulated through multiple signaling pathways involving the bone morphogenetic protein, transforming growth factor, activin, nodal, Wnt, rho-associated coiled-coil kinase, and fibroblast growth factor. Therefore, it is difficult to improve the currently saturated efficiency of cardiomyocyte differentiation by using novel molecules. Furthermore, it is hard to demonstrate the exact mechanism and interaction between novel cardiogenic molecules and other cardiogenic signaling pathways in these protocols. Indeed, recently published reports regarding cardiomyocyte differentiation from PSCs showed novel cardiogenic genes or markers, not cardiogenic molecules. In the near future, we plan to further investigate the effect of Ech A as a treatment for cardiomyocyte differentiation from human PSCs by using established high efficiency protocol.

Although adult stem cells (mesenchymal stem cells, cardiac resident stem cells, bone marrow derived stem cells, and endothelial progenitor cells) had modest effects in previous clinical trials, recent preclinical data shows there are promising when PSC-derived cardiomyocytes are implanted into infarcted primate myocardium [31]. Furthermore, implantation of cardiac cell sheets by using PSC-derived cardiomyocytes will firstly be tried in patients in the near future. Therefore, the cardiomyogenic effect of Ech A from our data is meaningful in terms of cardiac regenerative therapy by using PSC-derived cardiomyocytes that follow current trends. Therefore, the clinical importance of our study is that we firstly discovered the cardiomyogenic effect of Histochrome, a marine natural product derived drug with proven clinical safety. In addition, the finding of PKCι as the novel molecular target of Ech A could help for the repositioning of this drug for various diseases.

## 4. Materials and Methods

### 4.1. Maintenance of mESCs

The mESC line EMG7, which has an αMHC promoter-driven enhanced GFP gene, was maintained in 0.1% gelatin-coated 60-mm culture dishes without feeder layers, as previously described [14,19].

### 4.2. Induction of mESC-Derived Cardiomyocyte-Like Cell Using EBs

The hanging drop method was used to generate EBs from 750 cells in 20 μL differentiation media (differentiation day 0, D0). On day 4 of differentiation, EBs were seeded into 1% gelatin-coated culture dishes for each experiment. During differentiation, the culture medium was changed every day. Beginning on day 6 of differentiation (2 days after plating), EB growth was monitored daily for possible self-beating activity. For analyses, EBs were dissociated with 0.1% collagenase (Worthington, Lakewood, NJ, USA) plus 0.25% trypsin-EDTA (Gibco, Seoul, Korea) for 30 min at 37 °C [19].

### 4.3. Cell Viability Assay

Cells were seeded at 1 × 10^4^ cells/well in 96-well tissue culture plates. Ech A was dissolved in phosphate buffered saline (PBS) and PBS was treated as a control vehicle. After 24 h of incubation, the cells were treated with 0, 0.1, 1, 3, 5, 10, 30, or 50 μM Ech A for 24 h. Cell viability was measured using a quantitative colorimetric assay with MTT [3-(4,5-dimethylthiazol-2-yl)-2,5-diphenyltetrazolium bromide]; (Sigma-Aldrich, Gyeonggi-do, Korea). The optical density of formazan was measured at 570 nm using a microplate reader (Molecular Devices, San Jose, CA, USA) [12].

### 4.4. Quantitative Real-Time Polymerase Chain Reaction (PCR)

Total RNA was purified using the RNeasy mini kit (Qiagen). Complementary DNA (cDNA) was synthesized in a 50 µL volume using Super-Script™ III First-Strand Synthesis reagents (Seoul, Korea, Invitrogen). The cDNA was amplified using *Taq*DNA polymerase (Invitrogen, Seoul, Korea) in the presence of 1 μM oligonucleotide primers. The quantitative real-time PCR was performed using the iQ5 real-time system and the iQ SYBR™ Supermix (BioRad, Seoul, Korea). Expression of the target mRNAs relative to housekeeping gene expression (GAPDH mRNA) was calculated using the threshold cycle (C_T_) as *r* = 2^–Δ(ΔCT)^, where Δ C_T_ = C_T target_ − C_T GAPDH_ and Δ(Δ C_T_) = Δ C_T D8_ − Δ C_T D0_. The primer sequences are shown in Table 2 [19].

### 4.5. Western Blot Analyses

Cells were homogenized in a protein lysis buffer and centrifuged at 10,000× *g* for 10 min at 4 °C. After centrifugation, protein samples (50 μg) were transferred to nitrocellulose membranes, which were incubated with primary antibodies against sarcomeric α-actinin (Sigma-Aldrich), cTnT (Abcam, Cambridge, MA, USA), or β-tubulin (Abcam). Immunoreactive protein bands were detected using the SuperSignal™ West Pico system (The Thermo Scientific™, Seoul, Korea) and visualized using LAS-3000 PLUS (Fuji Photo Film Company, Tokyo, Japan) [19].

### 4.6. Immunocytochemistry

Differentiated EBs (D8) were cultured on gelatinized 12-mm glass cover slips, fixed for 30 min at room temperature in 4% paraformaldehyde, and permeabilized for 30 min at room temperature using 0.2% Triton X-100. After permeabilization, the EBs were blocked for 10 min at room temperature with CAS-Block solution (Invitrogen), and incubated overnight at 4 °C with mouse monoclonal antibodies against sarcomeric α-actinin (Abcam), cardiac troponin T (cTnT, Abcam), or connexin 43 (Cx43, Abcam). Additionally, goat anti-mouse IgG (Abcam) was used as the primary antibody to label EBs. Confocal images were obtained using a Carl Zeiss LSM 700 laser-scanning microscope (Zeiss, Oberkochen, Germany) [19].

### 4.7. Flow Cytometry Analyses

The percentage of cardiomyocyte-like cells in differentiated EBs was analyzed using αMHC-GFP. The cells were stained after dissociation of EBs with the MitoTracker™ RedCMXRos probe, TMRM, or MitoSox™ Red (Invitrogen) for 30 min at 37 °C to detect the mitochondrial membrane potential and the mitochondrial reactive oxygen species. To measure mitochondrial Ca^2+^levels, Rhod2-AM (Invitrogen) was incubated with cells for 2 h on ice, followed by incubation for 30 min at 37 °C. After staining, the cells were washed in PBS and analyzed using a dual laser FACS Calibur instrument (Becton Dickinson, Seoul, Korea) [19].

### 4.8. Kinase Activity Assay

A cell-free direct kinase activity assay was performed to identify the target kinases of Ech A that regulate cardiomyocyte differentiation from mESCs. The entire assay was performed using the Kinase Profiler^™^ Service (Eurofins Pharma Discovery Services, Aston Birmingham, UK) [32]. Individual kinase assay protocols are described in the Supplementary Materials. The dose-dependent inhibition of the PKCι activity was determined in the presence of 0, 0.5, 1, 5, 10, 50, 100, 500, or 1000 μM of Ech A.

### 4.9. Computational Protein-Ligand Docking Simulation

Docking calculations were performed using DockingServer (www.dockingserver.com) [33]. Gasteiger partial charges were added to the ligand atoms. Nonpolar hydrogen atoms were merged, and rotatable bonds were defined. Docking calculations were performed on the human PKCι kinase domain (PDB:3A8W) protein model. AutoDock tools were used to calculate essential hydrogen atoms, Kollman united atom type charges, and solvation parameters [34]. The van der Waals and electrostatic terms were calculated using the AutoDock parameter set- and distance-dependent dielectric functions. The Lamarckian genetic algorithm and the Solis & Wets local search method were used for docking simulations [35,36].

### 4.10. Surface Plasmon Resonance Binding Assay

The interactions between Ech A and its direct target kinase, PKCι, were determined using a dual channel surface plasmon resonance (SPR) instrument (SR7500DC; Reichert, Depew, NY, USA). The target full-length protein, PKCι (Abcam), was immobilized to the sensor chip surface by free amine coupling with a mixture of 0.1 M 1-ethyl-3-(3-dimethylaminopropyl) carbodiimide hydrochloride and 0.05 M N-hydroxy succinimide injection, followed by quenching the remaining activated carboxyl groups with 1 M ethanolamine, pH 8.5. A second reference cell was treated similarly without proteins. Ech A was prepared in a running buffer (0.01% PBS-T) and injected for 3 min (association time) at a flow rate of 30 μL/minute, followed by a dissociation phase of 3 min. Nonspecific background binding was subtracted from each sensogram using the SPR_V4017 Data Acquisition and Alignment programs (Reichert). Binding rates and constants were independent of the flow rate over a wide range of concentrations. The best-fit kinetic parameters were obtained by global fitting analyses using Scrubber2 (Biologic Software, Canberra, Australia) [37].

### 4.11. Statistical Analyses

Values presented are the mean ± standard error of the mean (SEM). Data were analyzed using the one way ANOVA and unpaired Student’s *t*-test. A value of *p* < 0.05 was considered statistically significant.

## 5. Conclusions

The marine bioproduct, Ech A, is a natural molecule with a potential for enhancing cardiomyocyte differentiation from mESCs via direct binding to PKCι and inhibition of its activity. In addition to its known beneficial cardioprotective effects, Ech A is a promising multifaceted marine drug for the treatment of cardiovascular diseases.

## Figures and Tables

**Table 1:** Computer-simulated Ech A interaction residues of PKCι kinase domain.

Residue No	Amino Acid	Interaction Type
392	LYS	Polar
395	LEU	Hydrophobic
399	ASP	Polar
400	THR	Hydrogen bonds, polar
401	THR	Hydrogen bonds, polar
402	SER	Other
420	ASP	Polar

**Table 2:** Quantitative real-time PCR primer sequences.

Gene	Primer Sequences
*α-actinin*	Forward 5′-AGCCAGGAACAGATGAACGA-3′ Reverse 5′-AAGTCGATGAAGGCCTGGAA-3′
*α-MHC*	Forward 5′-GCCCAGTACCTCCGAAAGTC-3′ Reverse 5′-GCCTTAACATACTCCTCCTTGTC-3′
*cTnI*	Forward 5′-CGTGGAAGCAAAAGTCACCA-3′ Reverse 5′-GTCCTCCTTCTTCACCTGCT-3′
*cTnT*	Forward 5′-CAGAGGAGGCCAACGTAGAAG-3′ Reverse 5′-CTCCATCGGGGATCTTGGGT-3′
*Cx43*	Forward 5′-ACGGCAAGGTGAAGATGAGA-3′ Reverse 5′-GAGAGACACCAAGGACACCA-3′
*Mef2c*	Forward 5′-ACCAGGACAAGGAATGGGAG-3′ Reverse 5′-GGCGGCATGTTATGTAGGTG-3′
*MLC2*	Forward 5′-GGCACCAAAGAAAGCCAAGA-3′ Reverse 5′-GGACCTGGAGCCTCTTTGAT-3′
*GAPDH*	Forward 5′-CACCATCTTCCAGGAGCGAG-3′ Reverse 5′-CCTTCTCCATGGTGGTGAAGAC-3′

## Supplementary Materials

The following are available online at http://www.mdpi.com/1660-3397/16/6/192/s1. Supporting materials for protocols for kinase activity assay.
